# Nanotheranostics-based Management of Head and Neck Cancer

**DOI:** 10.7150/ntno.81724

**Published:** 2023-02-05

**Authors:** Vivek P. Chavda, Pankti C. Balar, Srushti B. Patel

**Affiliations:** 1 Department of Pharmaceutics and Pharmaceutical Technology, L.M. College of Pharmacy, Ahmedabad, India.; 2 Pharmacy Section, L.M. College of Pharmacy, Ahmedabad, India.; 3 Pharmacy Section, Government Pharmacy College, Gandhinagar, India.

**Keywords:** Head and neck cancer, nanotheranostics, treatment, nanoparticles

## Abstract

Head and neck cancer is affecting a large sort of population. Many treatments are available on a regular base, but they have their limitations. Diagnosis in the early stage is essential to cope with the disease which is a limitation in the majority of present diagnostic tools. Many of them are invasive methods that lead to patient discomfort. Interventional nanothernostics is an emerging field in the management of Head and Neck cancer. It facilitates both diagnostic and therapeutic approaches. It also helps with the overall management of the disease. This method allows the early and accurate detection of the disease which improvises the chances of recovery. Additionally, it makes sure that the medicine is delivered specifically to increase clinical outcomes and reduce side effects. The use of radiation in addition to the medicine supplied can produce a synergistic effect. It contains several nanoparticles, including silicon and gold nanoparticles. This review paper focuses on the shortcomings of existing therapeutic techniques and how nanotheranostics fills the void.

## Introduction

Head and neck cancer (H&N) is the 7^th^ most common cancer type worldwide. It accounts for about 1.5% of death caused by cancer 1. In 2019, a total of 53,000 new H&N cancer cases (38,140 in males and 14,860 in females) and 10,860 HNC-related deaths (male-7,970, female-2,890) were observed in the USA. Additionally, WHO estimates that by 2030, there will be 439,000 mouth and oropharynx cancer cases. Concerning stage at diagnosis, approximately 20% of cases fall into the distant stage cancer category, 29% of cases fall into the localized category, and 47% fall into the regional category 2,3. Across the globe, depending on the site of the tumor and grade, the overall five-year survival rate for HNC is approximately 45% to 60%. The diagnosis of cancer in the advanced stage has the least treatment outcome than the early stage along with survival rate. However, with the advancement in the diagnosis and treatment modalities improvement has been seen in the survival of HNC patients, irrelevant of the stage. In addition, chemotherapy along with radiotherapy contribute about a 6.5% increment in the last five-year survival rate 3-5. H&N usually initiates with the squamous cell present in vivid organs such as the oral cavity, larynx, pharynx, nasal, cavity and salivary gland. The prominent site of the tumor varies with geography, which further transmits to the lymph node if the cancer mass metastasis. The disease is usually curable, provided detected in the early stage. Early detection is a hurdle because of a very minute cell mass in the initial development of the disease. Biomarkers such as PDL-1, Interferon-γ gene mutation, and many others are available but they are incorporated with possibilities of false results 6. Radiotherapy is the preferred method for the treatment of H&N but the ongoing trails state that the outcomes are not promising 7. Even after the detection of the disease, it is crucial to manage the disease in a manner to increase the survival rate of the patients with the least discomfort.

This review focuses on the prevalence of H&N cancer, factors that could influence directly or indirectly to its occurrence, and how nanotheranostics proves to be a boon in the management of the disease.

## Occurrence

There are numerous factors that contribute to the occurrence of the disease such as alcohol consumption, infection with human papillomavirus (HPV) (especially HPV16and HP18), Epstein-Barr virus (EBV) exposure to radiation, hereditary, genetic disorder, sex and many more. According to estimates, among these, mostly 60%-70% of oropharynx cases are caused by the HPV virus, because of the increase in the prevalence of the HPV virus. A study concluded that the prevalence of H&N is higher in men (12.42 per one lakh) as compared to females (3.71 per one lakh) 8. A study proposed that high oral HPV infection in man leads to H&N in higher concentration [9,p.2009-2010]. Both the sexes are highly susceptible to oral cancer. Men have high tendency to develop larynx cancer subsequently followed by hypopharynx wherein females develop salivary cancer easily followed by oropharynx cancer. Occurrence of laryngeal cancer and hypopharyngeal cancer is firmly associated with smoking and alcohol consumption, respectively 10,11. In the terms of geographical variation in Asia, nasopharyngeal cancer and mouth cancer are more prominent in Hong Kong and India wherein laryngeal cancer and pharyngeal cancer are common in other regions 12,13. Because of the outbreak of HPV in African countries, the occurrence and mortality are higher than in African males 14. Median age of diagnosis of viral (HPV and EBV) and non-viral associated H&N is 53 years (for HPV and 50years for EBV) and 66 years, respectively. With this highly diversified profile of occurrence, the diagnosis and treatment became a hurdle in management of the disease.

## Complication

Although many researches and trials are incorporated to get better understanding of the disease, it still remains a mystery. H&N is a hazardous disease which demands for prompt treatment and diagnosis to amplify the chances of fastened recovery. This helps both patient and physician to adopt to the situation and come up with better solution. Hurdles in the management of disease questions the health care system and needs an urgent replacement.

### Diagnosis

Even with a developed system of diagnosis of other crucial disease, H&N remains in a limitation of it. No screening methods are present for the diagnosis, and careful physical examination is demanded. Standard methods (invasive method) are employed to ensure the station of the disease, but the pathologist faced the issue in the methods due to lack of sensitivity to minute changes. Another approach to detect the disease is through non-invasive methods i.e., Exhaled breath analysis 15. Though being effective in the diagnosis, the sample collection method is sophisticated and the result relies on many contributing factor 16. Detecting the disease by biopsy is still doubtful and requires appropriate validation 17.

### Treatment

The current methods involved in the treatment of H&N are surgery, chemotherapy, immunotherapy, radiation therapy and multimodality approaches. In the past few decades, the multimodality approaches are recognized as standard care treatment for patient who is diagnosed at the advanced stage of HNC, which accounts for 60% of HNC cases. These approaches mainly include chemotherapy along with radiation therapy. While early-stage (I or II) cancer can be effectively managed by surgery or radiation therapy 18. When the lymph node is removed with the invasive technique, it reduces the flow of lymph and hence get accumulated in the tissues which can cause a chronic swelling. Other side effects include weight loss, lymphedema, difficulty in eating and many more 19. The FDA has approved monoclonal antibodies against the epidermal growth factor receptor (EGFR); however, they are not as effective radiosensitizers as cisplatin. Proton beam therapy is some of the widely accepted therapeutic approaches which involves radiotherapy. The pitfalls of these therapy consist of dermatitis, neurologic toxicity, need of feeding tube and many more which limits the quality life of surviving patients 20.

Due to the difficulties in both diagnosis and treatment, it is necessary to take a creative approach to managing the condition and enhancing the quality of life for survivors. Nanotheranostics is an emerging field, if discovered in depth, can come up with a complete new set of drug delivery which ensures early detection, targeted drug delivery, least adverse event and many more add-ons.

## Interventional nanothernostics in Head and Neck cancer

**Figure 1:**
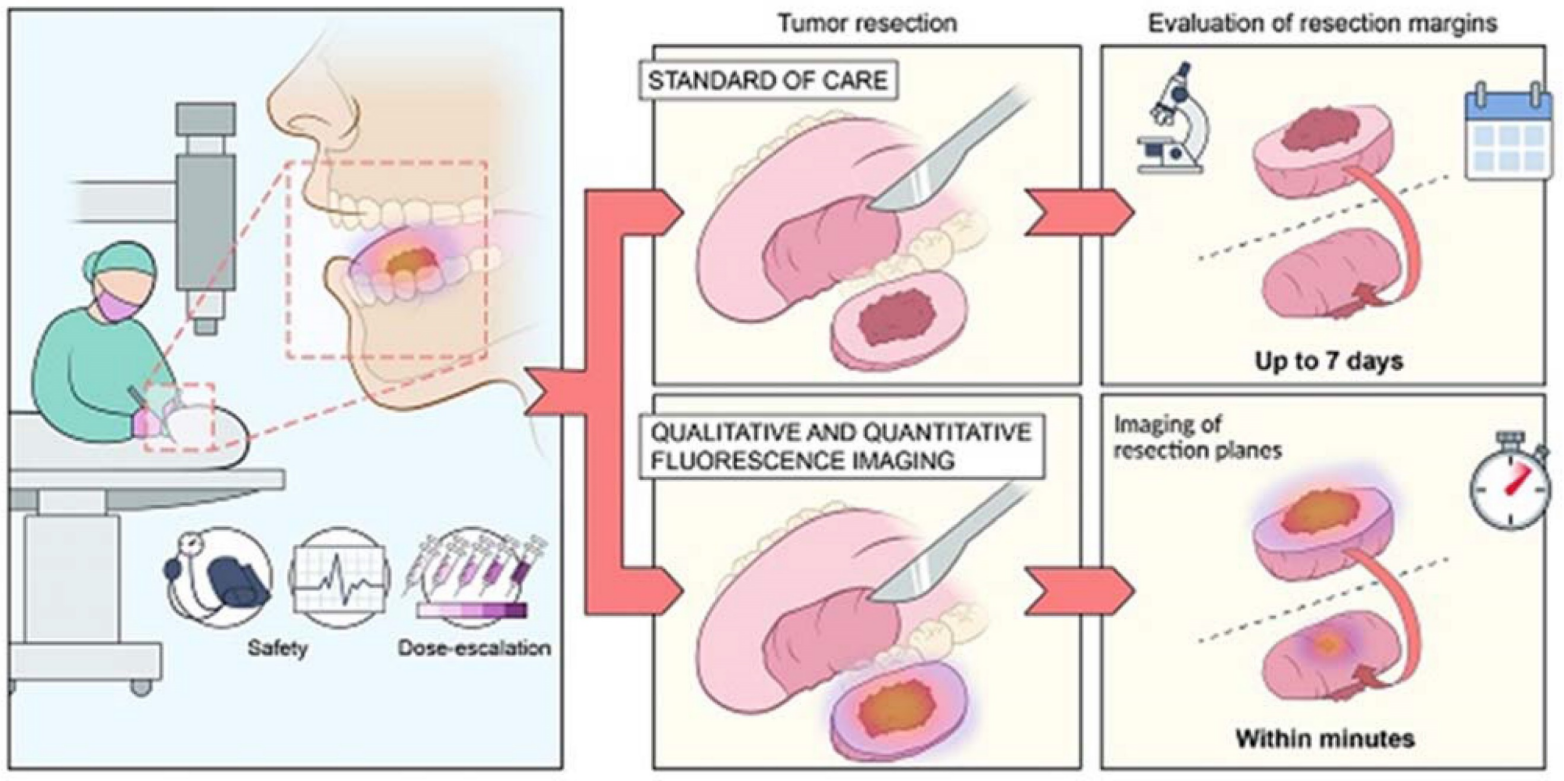
** Tumor resection is a conventional method used for the removal of benign tumor in H&N cancer while with the help of interventional nanotheranostics,** it becomes a lot easier process. It provides both qualitative as well as quantitative evaluation of the tumor through fluorescence imagining. It targets the specific cell mass which helps to deduct the unwanted effect. For the diagnosis as well, where the normal evaluation methods take more than a week to confirm the outcomes, theranostics does the same job with higher efficacy and accuracy within few minutes. Adopted by CC by 4 from 22.

Today in every field interventional nanotheranostics applications are being studied because of their immense beneficial properties especially, in cancers. Generally, cancers are diagnosed in late phases and poor treatment outcomes make it difficult to treat and cure completely. However, Interventional nanotheranostics involves the use of nanoparticles to solve the diagnosis and treatment obstacles of conventional therapies and ultimately increases survival rate 21. A comparative presentation of the traditional therapy approach and novel nanotheranostics is explained in Figure 1.

**Figure 2:**
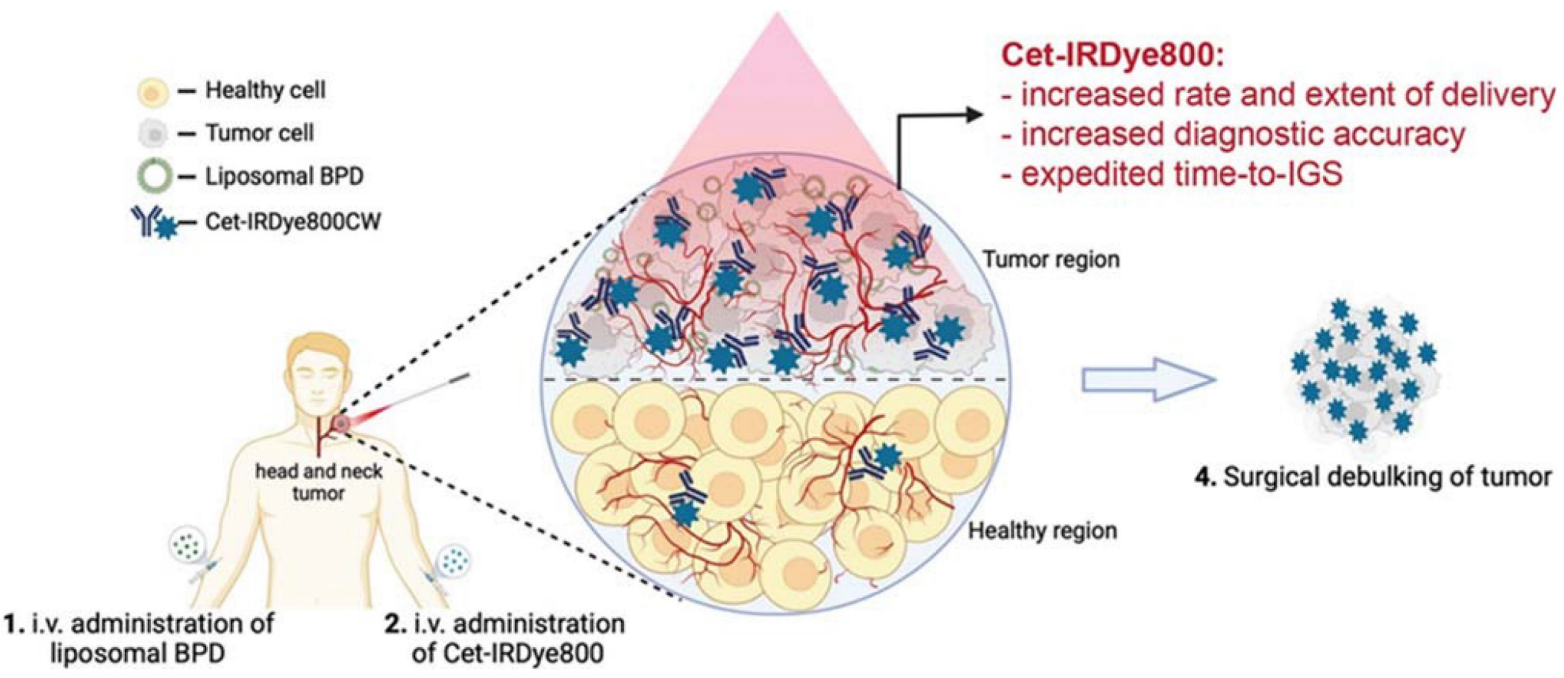
** Administration of Cet-IRDye800 associated with the liposomal molecules leads to the nanotheranotics drug delivery system.** It helps the drug molecule to target the tumor mass. There is very low concentration of drug in normal cells which helps to reduce the adverse events of drugs. Cet-IRDye800 has dual action of therapeutic as well as diagnostic(targeting). This conjugated molecule increases the rate of delivery along with the extent of delivery. It increases the diagnostic accuracy of the molecule. This approach helps to remove the tumor through the surgical method after detecting its accurate position. Adopted by CC by 4 from 29.

Head and neck cancer (HNC) mortality and morbidity rates are on the rise in part due to late detection and major therapeutic restrictions. Moreover, patients of HNC require staging evaluations, effective treatment, and after-treatment monitoring at regular intervals 23. For treatment of HNC nanoparticles (NPs) like Metallic, metal oxides, and polymeric NPs are used. The small size of nanoparticles (NPs) and the remarkable ability to interact with biological molecules make its greater use in cancer interventions 24. NPs are available in a diameter range of 1 -100 nm and can act as a carrier that can carry therapeutic agents, targeting ligands or diagnostic agents. Utilizing NPs as a drug carrier and a wide range of physicochemical as well as optical and biological properties improves drug delivery by targeting tumor sites 25,26. The most critical properties of NPs are particle size and size distribution as they directly impact drug distribution. The NPs with small sizes can easily penetrate the tumor microenvironment. While large-sized particles are not easily cross the tumor microenvironment but they have more capacity to incorporate drug load and can slowly release drugs at the active tumor site in a controlled manner. Hence, according to the need particle size can be adjusted. Similarly, it allows other modifications like attachment of targeting ligands, polymer coating on nanocarriers, surface charge development, and drug payload to maximize efficacy and reduce toxicity through controlling factors like solubility, permeability, drug distribution, bioavailability, etc. 26-28. Targeted delivery of the drug molecule is the unique property of the nanotheranostics drug delivery system. It is pictorially explained in Figure 2.

**Figure 3:**
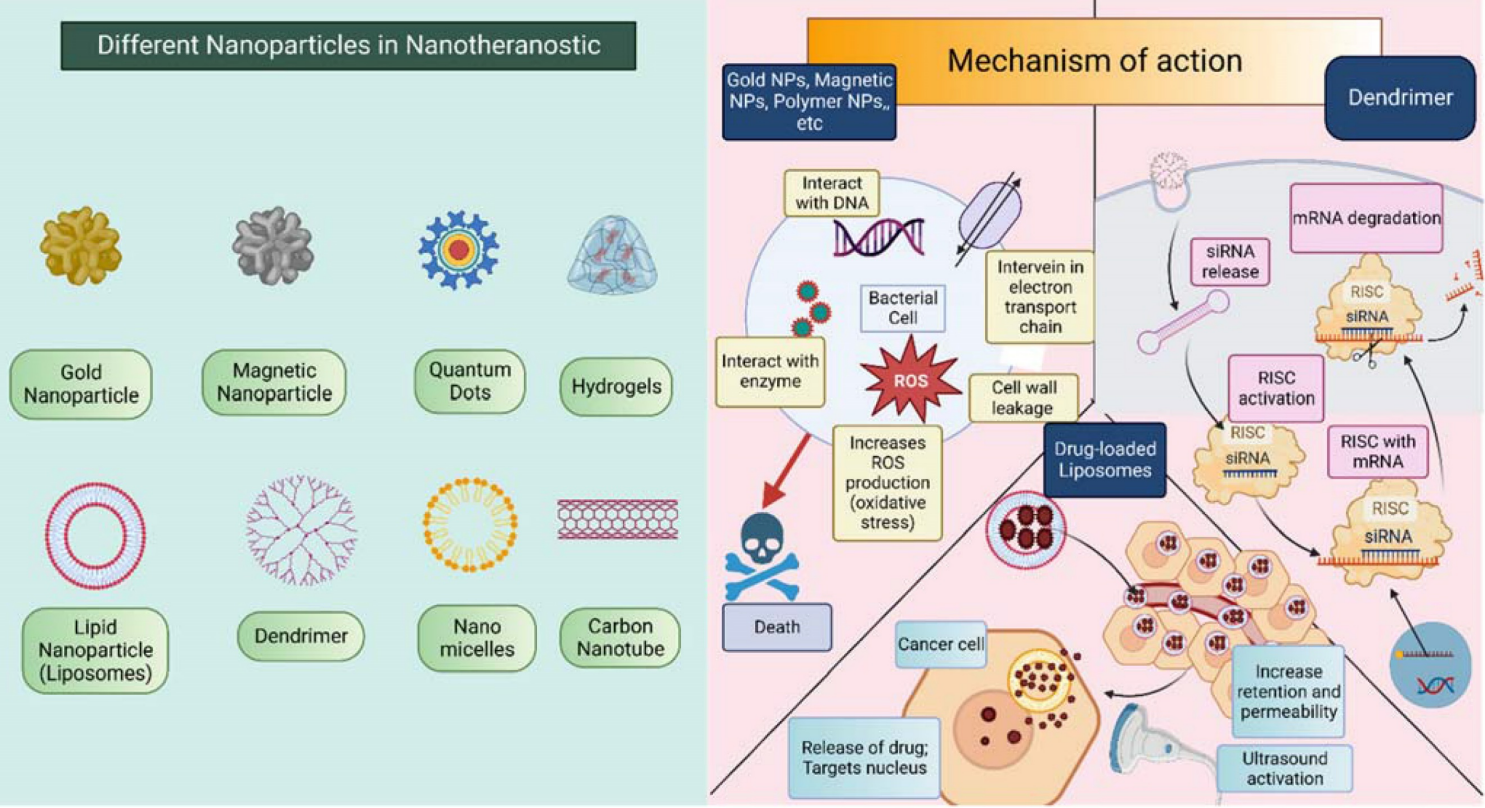
** Different nanoparticles that are incorporated in the theranostic approach of H&N cancer.** Mechanism of action of nanoparticle differs for different nanoparticle. This figure is created in Biorender.com.

NPs have two main ways of showing their effects: active targeting and passive targeting. In theranostics, NPs can use any of these two methods. The earlier approach makes use of the tumor vasculature's increased permeability and retention (EPR) effect. The damage of tumor blood vessels increases the permeability and also impaired the lymphatic drainage system which provides a way for NPs to easily penetrate the tumor microenvironment and can directly act on cancerous cells effectively due to the accumulation of the drug in high concentration 30. The second mechanism by which NPs exhibit their effect is active targeting also called ligand-mediated targeting. In this method, the surface of NPs is modified by attachment with ligands such as proteins, enzymes, antibodies, peptides, etc. these ligands bind with only specific substrates in the body i.e., selectively bind with cancer cells which improve therapeutic response and reduce toxicity to healthy cells 31-34. Refer Figure 3 for pictorial presentation of NPs along with detailed mechanism of action corresponding to NPs. Apart from this, they also exert responses to external or internal stimuli like temperature, pH, reactive oxygen species (ROS), etc. 35 the stimuli-responsive nanoparticles especially endogenous stimuli-responsive NPs show greater biocompatibility, fewer side effects, and better therapeutic outcomes. Moreover, it also can be modified to make a multifunctional drug delivery system for active targeting, combination therapy, and for interventional theranostic purposes 35,36.

Nowadays, NPs are most frequently used in the treatment of HNC like radiotherapy, chemotherapy, immunotherapy, and other combination therapies like hyperthermia, photodynamic therapy, etc. as they overcome the challenges of traditional therapies. Various NPs that are used as nanocarriers in the treatment as well as diagnosis of HNC. Some examples of NPs are Metal NPs such as Gold NPs (AuNPs) and Gadolinium NPs, Magnetic NPs, Polymer-coated NPs, Lipid-based NPs, Nano micelles, Microemulsions, Dendrimers, Quantum dots, etc. 28,37. Among these, gold NPs are most widely used as radiosensitizers. In traditional radiation therapy, resistance to radiation is a major challenge. To overcome this dilemma and to improve radiation efficiency, AuNPs are recognized, as they have a higher atomic number (Z=72), higher radiation absorption capacity, adaptability, and some other physicochemical properties such as good biocompatibility, long circulation time, etc. The core concept behind the AuNPs as radiosensitizers is that they have a high atomic number which absorbs less radiation energy itself but around the gold particles more radiation energy is deposited in the tumor microenvironment which leads to cell death of cancerous cells. It enhances the radiation therapy efficacy and reduces the toxicity to normal tissues 23,38-40. In addition, AuNPs also have Surface Plasma Resonance (SPR) properties which are useful in photothermal therapy. Upon electromagnetic radiation (EMR) on AuNPs, they resonate to enhance absorption and scattering of light which will further produce hyperthermia (increased temperature, near 45 °C) due to EMR and shows cell necrosis. Preferentially infrared radiation, nearly 700-1000nm is used in this therapy 41,42. Clinical findings on AuNPs show that the synergistic effect can be produced by combining chemotherapy and radiotherapy if the surface of NPs is incorporated with a polymer like Polyethylene glycol (PEG) or modified with ligands like proteins, enzymes, or antibodies. It also reduces the unwanted distribution of AuNPs. However, still more research is required on cellular uptake and non-specific biodistribution to make radiation therapy effective 42-44. From the metal and metallic oxide category other NPs like Iron Oxide, Gadolinium, Silver, and Cerium Oxide NPs are also used in the treatment of HNC.

The other most commonly applicable NPs in the diagnosis and treatment of HNC are liposomes or lipid-based NPs. The spherical liposomes are made up of phospholipids and cholesterol bilayer and an aqueous core part. It makes liposomes amphiphilic which allows encapsulation of hydrophilic as well as hydrophobic drugs in the formulation. Hydrophilic drugs such as doxorubicin are mainly trapped in the aqueous part while hydrophobic drugs such as paclitaxel, and amphotericin B are trapped in a phospholipid bilayer. Liposomes can be classified according to size and structure into the following class: Large Unilamellar Vesicles (LUV), Medium Unilamellar Vesicles (MUV), Small Unilamellar Vesicles (SUV), Multilamellar Vesicles (MLV), Oligolamellar Vesicle (OLV), And Unilamellar Vesicles (UV). Liposomes come in contact with different proteins and cell components which may destabilize the liposome formulation. Thus, cholesterol is added as a stabilizer to enhance the stability of the formulation. Moreover, its advantages like easy preparation methods, higher drug payload in formulation, non-toxic, biocompatibility, biodegradability, and, facile cell membrane penetration may enable improved chemo- and radiotherapy efficiency by enhancing drug biodistribution and pharmacokinetic characteristics and increasing drug accumulation in tumor cells of HNC 28,45-48. Besides the wide use of liposomes and AuNPs, various polymeric NPs are also at the top of the list. Chitosan NPs, Hyaluronic acid NPs, Poly (lactic-co-glycolic Acid) NPs, and Polyethylene Glycol are examples of Polymeric NPs. Different synthetic, semisynthetic, and natural polymers are used to formulate polymeric NPs which can facilitate targeted and controlled drug delivery systems however, drug resistance limits its use 49,50.

As the technology expanded, the treatment of HNC also has been explored. Earlier we mentioned that there are numerous treatment modalities options available like surgery, radiotherapy, chemotherapy, photodynamic therapy (PDT), photothermal therapy (PTT), etc. nowadays, PDT has gained more attention because of reduced side effects and better selectivity. Also, it is a nonsurgical method that enhances patient compliance too. In this treatment, light source and photosensitizing agents (PSs) are used; the light source falls on photosensitizer at the tumor targeting site which produces the cytotoxic effect on the cancerous cells by producing reactive oxygen species (ROS)^51^,^52^. Various light sources have been invented to use in PDT such as near-infrared light (NIR), X-ray light, and internal and interstitial light but light penetration depths with these sources are a bit challenging therefore, these are still under research. However, some new sources like microwaves, radio waves, ultrasound, electrical fields (EF), and magnetic fields are underneath innovation for the efficient excitation of the photosensitizing agent. PDT therapy is still unlikely used in clinical applications as it also faces hurdles with proper selection and use of PSs at the specific tumor site. Substantial work must be done to enhance its stability, targeting ability, solubility, and biodistribution in case, if PSs used orally. PSs can be applied topically, injected intravenously or intraperitoneally, and can be orally administered. The challenges that we are fronting with PSs can be decreased to some extent by incorporating PSs with nanoparticles or by encapsulating them into nanocarriers. This nanoformulation protects the PSs from the outer environment, increases bioavailability and site specificity, and decreases drug inactivation 53-55. Apart from PDT various other experimental approaches also investigate HNC such as gene therapy, immunotherapy, hyperthermia, etc.

### Clinical trials

The very first step to access the safety, efficacy, and toxicity of any compounds preclinical trials are conducted on animal models such as carcinogen-induced animal models of oral cancer (to study histopathological features of oral mucosa in cancer), genetically engineered mouse models (to study significant biological consequences of mutation), Patient-derived xenograft models (PDX). PDX models more closely resemble human cancer as it includes the implantation of tumor tissue or cells into an immunocompromised or humanized mouse and the natural growth of cancer is allowed 56,57. If reliable and promising results are achieved then the further actual clinical study is designed and performed on human volunteers, to evaluate the pharmacokinetic and pharmacodynamic profile of a drug. The potential application of NPs is increasing day by day in the field of HNC treatment and diagnosis. Thus, to provide accurate and effective interventional nanotheranostics along with reduced toxicity, various clinical research has been conducted on NPs which are listed in the below table.

From the above-mentioned clinical data, most of the clinical trials that are completed or still ongoing in phase 1 or phase 2 are designed to evaluate NPs application in the treatment and diagnosis of HNC. None of the trials reached phase 3. It is observed that paclitaxel-albumin NPs are widely studied for radiation therapy in most clinical trials. Cetuximab, cisplatin, and Hafnium Oxide NPs are also assessed for their use in HNC.

## Current hurdles and future prospects

Despite having a wide range of advantages and applications, nanoparticles still confront difficulties in many stages thus, NPs should be carefully studied in preclinical and clinical studies. The major challenge is NPs are rapidly eliminated or encountered by the immune system of a patient. So, the drug action time is limited while nanomaterials that are used to carry a drug (carrier) remain present which may cause toxicity. However, a targeted drug delivery system may help to improve the efficacy of treatment 58. other areas in which we face challenges are the route of administration, drug distribution, biological barriers, preparation methods, pharmacological effects prediction, stability of NPs formulations, etc. 59. At last, it can be said that NPs are in their raising stage and further many more studies are required to expand the field of nanotheranostics in the management of cancers.

## Conclusion

H&N is of great concern to healthcare system and needs an undivided attention for the development of better management strategies. Many diagnostic methods are available but they are not up-to a mark both in efficacy and patient reliance. Similarly, therapeutic approaches need a new bench mark which is provided by nanotheranostics. It is a novel method to address both therapeutic as well as diagnostic approaches. It ensures the targeted delivery which reduces the interaction with normal cell. It also facilitates the additional therapy to target the specific tumor cells. Being such a miraculous drug, it still lacks the detailed experimentation. The article interrogates to explore this methodology in detail to improvise the healthcare sector.

## Figures and Tables

**Table 1:** clinical studies data on the treatment of HNC with interventional nanotheranostics.

Study Ids	Status	Phase	Study Start	Study Completion	Remark	NCT Number
08-084	Completed	Phase 1	August 2008	August 2016	Nab-paclitaxel + cetuximab + intensity-modulated radiation therapy (IMRT) used 25 patients were involved Common AE observed was lymphopenia Efficacy for 2-year overall survival was found 91%	NCT00736619
STU 072010-046	Completed	Phase 1 Phase 2	March 1, 2009	August 3, 2015	Nab-paclitaxel, cisplatin, and cetuximab are used 34 patients were involved	NCT00851877
BIND-014-001	Completed	Phase 1	January 2011	February 2016	Docetaxel is used as a drug moiety 52 patients were involved Recommended dose was 60 mg/m^2^	NCT01300533
201202113	Completed	Phase 2	August 9, 2012	October 31, 2019	Induction chemotherapy +cisplatin+ radiation therapy 30 patients were enrolled Swallowing, oral pain and voice quality were improved along with the therapeutic upliftment	NCT01566435
NCI-2012-02179	Active, not recruiting	Phase 1	March 26, 2013	December 2022	Nav-paclitaxel-based induction + nab-paclitaxel-based concurrent chemotherapy + re-irradiation 48 patients were involved Recommended dose was 100 mg/m^2^	NCT01847326
IRB17-0104	Active, not recruiting	Phase 2	June 27, 2017	July 2023	39 patients were enrolled Radiography+ systemic therapy	NCT03107182
NCI-2021-00122	Recruiting	Phase 2	April 7, 2021	September 30, 2026	NBTXR3, Radiation Therapy, and Pembrolizumab Administered in intertumoral/intranodal 60 participants involved	NCT04862455
SNB101P01	Unknown	Phase 1	October 21, 2020	March 31, 2022	36 participants enrolled Drug: SNB-101	NCT04640480
